# An in-silico study of the mutation-associated effects on the spike protein of SARS-CoV-2, Omicron variant

**DOI:** 10.1371/journal.pone.0266844

**Published:** 2022-04-21

**Authors:** Tushar Ahmed Shishir, Taslimun Jannat, Iftekhar Bin Naser

**Affiliations:** 1 Department of Mathematics and Natural Sciences, BRAC University, Dhaka, Bangladesh; 2 Rangamati General Hospital, Chattogram, Bangladesh; Russian Academy of Medical Sciences, RUSSIAN FEDERATION

## Abstract

The emergence of Omicron (B.1.1.529), a new Variant of Concern in the COVID-19 pandemic, while accompanied by the ongoing Delta variant infection, has once again fueled fears of a new infection wave and global health concern. In the Omicron variant, the receptor-binding domain (RBD) of its spike glycoprotein is heavily mutated, a feature critical for the transmission rate of the virus by interacting with hACE2. In this study, we used a combination of conventional and advanced neural network-based in silico approaches to predict how these mutations would affect the spike protein. The results demonstrated a decrease in the electrostatic potentials of residues corresponding to receptor recognition sites, an increase in the alkalinity of the protein, a change in hydrophobicity, variations in functional residues, and an increase in the percentage of alpha-helix structure. Moreover, several mutations were found to modulate the immunologic properties of the potential epitopes predicted from the spike protein. Our next step was to predict the structural changes of the spike and their effect on its interaction with the hACE2. The results revealed that the RBD of the Omicron variant had a higher affinity than the reference. Moreover, all-atom molecular dynamics simulations concluded that the RBD of the Omicron variant exhibits a more dispersed interaction network since mutations resulted in an increased number of hydrophobic interactions and hydrogen bonds with hACE2.

## Introduction

Coronavirus disease (COVID-19), caused by the severe acute respiratory syndrome coronavirus 2 (SARS-CoV-2) virus, rapidly spread throughout the world and was declared a global pandemic as a public health emergency of international concern, which continues to have serious negative effects on health and the economy worldwide [1, 2]. In order to halt the spread of SARS-CoV-2 virus, several vaccines have been developed and implemented, but some of these efforts have been hindered by mutations leading to new virus variants [3–5]. A recently emerged and rapidly spreading variant of SARS-CoV-2, named Omicron, has triggered worldwide concern, and the World Health Organization declared it a ’variant of concern (VOC)’ on November 26, 2021 [6, 7]. VOC is the name given to a SARS-CoV-2 virus variant with mutations affecting the spike protein receptor-binding domain that increases the binding affinity within the RBD-hACE2 complex and increase the viral transmissibility [8]. A total of 30 mutations have been identified on the spike glycoprotein of the Omicron variant, of which 15 are located on its receptor-binding domain, and some of these mutations are also present in other VOCs [6, 9, 10]. As of today, the Omicron variant is the most genetically diverse strain seen in a large number of cases, which may increase the risk of reinfection, greater disease transmission, the severity of the disease and escape from diagnosis while decreasing vaccine effectiveness.

A SARS-coV-2 infection begins with the binding of the viral spike protein to the Angiotensin-converting enzyme 2 (ACE2), followed by the proteolytic processing of the trimeric spike protein into S1 and S2 subunits by the serine protease furin [11, 12]. In turn, S2 aids in the fusion of the viral cell membrane with the host, leading to viral entry into the cell cytoplasm via endocytosis, where it blocks a number of antiviral pathways, eventually causing an upsurge in pro-inflammatory cytokine secretion and NF-*B activation that causes cell death and hyper-inflammation [13, 14]. Therefore, the mutations in the spike protein would have a very large impact on the virulence of SARS-CoV-2, transmissibility, as well as the efficacy of the current vaccines and therapies [15, 16]. Currently, vaccine or neutralization antibody programs are primarily focused on RBD-ACE2 interactions [17–19]. However, in addition to ACE2, a number of other host molecules are also reportedly involved in SARS-CoV-2 attachment to cells and act as entry factors [20–22]. Therefore, most, if not all, of the current antibody strategies may not inhibit SARS-CoV-2 virulence or reduce the hyper-immune response. Additionally, higher mass vaccination rate in many countries compared to others is increasing the risk of SARS-CoV-2 mutating into a new strain that might be resistant to current vaccines [23]. Highly infectious variants require greater vaccine penetration to build protective immunity therefore, it is vital to study the effect of the mutations on the immunologic properties for evaluating the current therapies and development of new vaccines to combat the SARS-CoV-2 pandemic.

Several studies have reported a higher affinity for the ACE2-receptor in the mutant spike protein of Omicron than in wild-type SARS-CoV-2, as well as a greater ability to evade the immune system, which might result in higher viral transmissibility [24, 25]. To the best of our knowledge, none of the studies examined detailed effects on physicochemical, structural, or immunologic properties of the spike protein due to the large number of mutations found in the spike protein. Therefore, the purpose of our study was to use an in silico approach to comprehensively analyze the spike protein of the Omicron variant with respect to the reference variant to distinguish the differences from physicochemical, structural, immunologic and functional perspectives. Additionally, we have conducted protein-protein docking with the ACE2 receptor in order to investigate the effect of mutations on protein interactions. Finally, we performed atomistic molecular dynamics simulations of the RBD-ACE2 complex of Wild-type SARS-CoV-2 and Omicron variants for a detailed molecular analysis.

## Methods and materials

### Data retrieval and annotation

SARS-CoV-2 reference spike protein sequence was obtained from UniPort with accession number P0DTC2 and the first complete Omicron genome from GISAID with accession number EPI_ISL_6640916 [26, 27]. The Omicron genome sequence was then annotated using the Cov-Seq program, followed by translation using the EMBOSS transeq tool [28, 29]. A pairwise alignment of spike protein sequences was later performed with Clustal Omega, followed by the analysis of mutations [30]. Furthermore, the PDB structure of the RBD-ACE2 complex with accession 6M0J and the PDB structure of the whole spike protein with accession 7N1U were used as reference, downloaded from RCSB PDB database [31].

### Physicochemical parameter analysis

The spike protein of the reference and Omicron variants were subjected to preliminary sequence analyses to distinguish their physicochemical differences. The amino acid composition, molecular weight, distribution of charged residues, hydropathicity, aliphatic index, instability index, and a few other parameters are calculated mainly using the online server EMBOSS Pepstats [29]. Further verification of the results from Pepstats was conducted using ProtParam, Prosite and AA-prop [32, 33]. In addition, another web server called VOLPES was used to compare and visualize residue-level physicochemical properties [34].

### Structural properties analysis

JPred4 was used to predict the secondary structure of spike proteins based on the JNett algorithm, which is one of the most advanced and accurate methods [35]. NetSurfP-2.0 tool was used to evaluate further the prediction, which utilizes convolutional and long short-term memory neural networks to predict secondary structure, solvent accessibility, and residual disorder [36]. Furthermore, the flDPnn server was used to predict intrinsically disordered regions and their functions in conjunction with NetSurfP-2.0 [37]. Following that, the PredyFlexy server was used to determine the flexibility of the structures, while consurf and predictprotein were used to predict conserved regions [38–40]. Finally, AlphaFold2 was used to predict the tertiary structure of the Omicron spike protein [41]. Following the prediction of 3D structure, we calculated the RMSD and TM-score between the predicted Omicron spike protein and our reference spike protein (7N1U) using the TM-align tool and calculated the overlap of common contacts using the CMView program [42, 43].

### Functional properties analysis

In order to determine the effect of mutations on protein stability, Dyanmut2 and DeepDDG were used, where Dyanmut2 used normal mode analysis and graph representations of protein structures, and DeepDDG used neural networks to predict the effect of mutations on protein stability [44, 45]. In addition, several tools, including SNAP2, PROVEAN and SIFT, were used to assess the impact of mutations on function [46, 47]. As a final step, we predicted how mutations would affect the propensity of SARS-CoV-2 to cause disease using the VarSite webserver [48]. Whenever more than one tool was employed to predict the effects, the common outcomes were taken into consideration.

### Immunologic properties analysis

First of all, CD8+ T-cell, CD4+ T-cell and B-cell epitopes were predicted in NetMHCpan-4.1, NetMHCIIpan-4.0 and BepiPred-2.0 servers [49]. To analyze the immunologic properties in detail, all the epitopes passing the default threshold value of the servers were considered for downstream analysis. Then, the immunogenicity of the CD8+ T-cell epitopes was predicted using the Class I Immunogenicity analysis tool and immunogenicity for CD4+ T-cell epitopes was checked using the CD4+ T-cell immunogenicity prediction tool of the IEDB database while immunogenicity of B-cell epitopes was predicted with the iBCE-EL server [50, 51]. Antigenicity, pro-inflammatory and anti-inflammatory potentials for all types of epitopes were predicted using VaxiJen v2.0, PIP-EL and AIPpred webservers respectively [52–54].

### Molecular docking and protein-protein interaction analysis

First, we retrieved the RBD-ACE2 protein complex with accession number 6M0J and separated the chains. Then, the Pymol mutagenesis wizard was used to introduce the specific mutations at the appropriate residues in the receptor-binding domain. After preparing the protein, Cluspro and HDOCK were used to dock the reference and Omicron RBD to the ACE-2 receptor (6M0J, chain A) [55, 56]. We then used the PRODIGY webserver to calculate the binding affinity and the PIC server to investigate the interactions between RBD and hACE2 [57, 58]. Finally, the Pymol graphical software was utilized for figure generation [59].

### Molecular dynamics simulation

All-atom MD simulation was carried out in GROMACS 2021.2 software package, and ACE2-RBD protein complexes of both reference and Omicron variants were prepared using the CHARMM36 force fields [60, 61]. Each protein complex was then solvated with the TIP3P water model by adding 0.15mM sodium chloride within a dodecahedron box. The distance between the protein complex and the corner of the box was set to 1.2nm. The system energy was minimized with the Steepest Descent algorithm in 50,000 steps, followed by the system was equilibration in two phases. Firstly, 10ps NVT (constant number of particles, volume, and temperature) simulation was performed to equilibrate the temperature at 310.15K guided by V-rescale temperature coupling algorithm, followed by 100ps NPT (constant number of particles, pressure, and temperature) simulation to equilibrate the system at 1atm pressure and 310.15K by using Parrinello-Rahman barostat algorithm [62]. Finally, the MD simulations of both reference and Omicron variant ACE2-RBD systems were run for 200ns with a time step of 2.0fs under NPT ensemble using GROMACS 2021.2 software and long-range electrostatic interactions were computed using Particle Mesh Ewald (PME) algorithm. The cutoff values of the electrostatic and Van der Waals interactions were set to 12 Å while the linear constraint LINCS algorithm was used to constrain all covalent bond lengths, including hydrogen. MD trajectories were analyzed using GROMACS’ integrated tools for computing root-mean-square deviation (RMSD) and difference root-mean-square fluctuation (RMSF). Lastly, we investigated hydrogen bond interactions and their relative frequencies with the VMD package, setting the hydrogen bond distance and angle to 3.0 Å and 20°, respectively, and calculated the binding energy using the Prime 3.0 MM-GBSA module [63].

## Results

**Fig 1:**
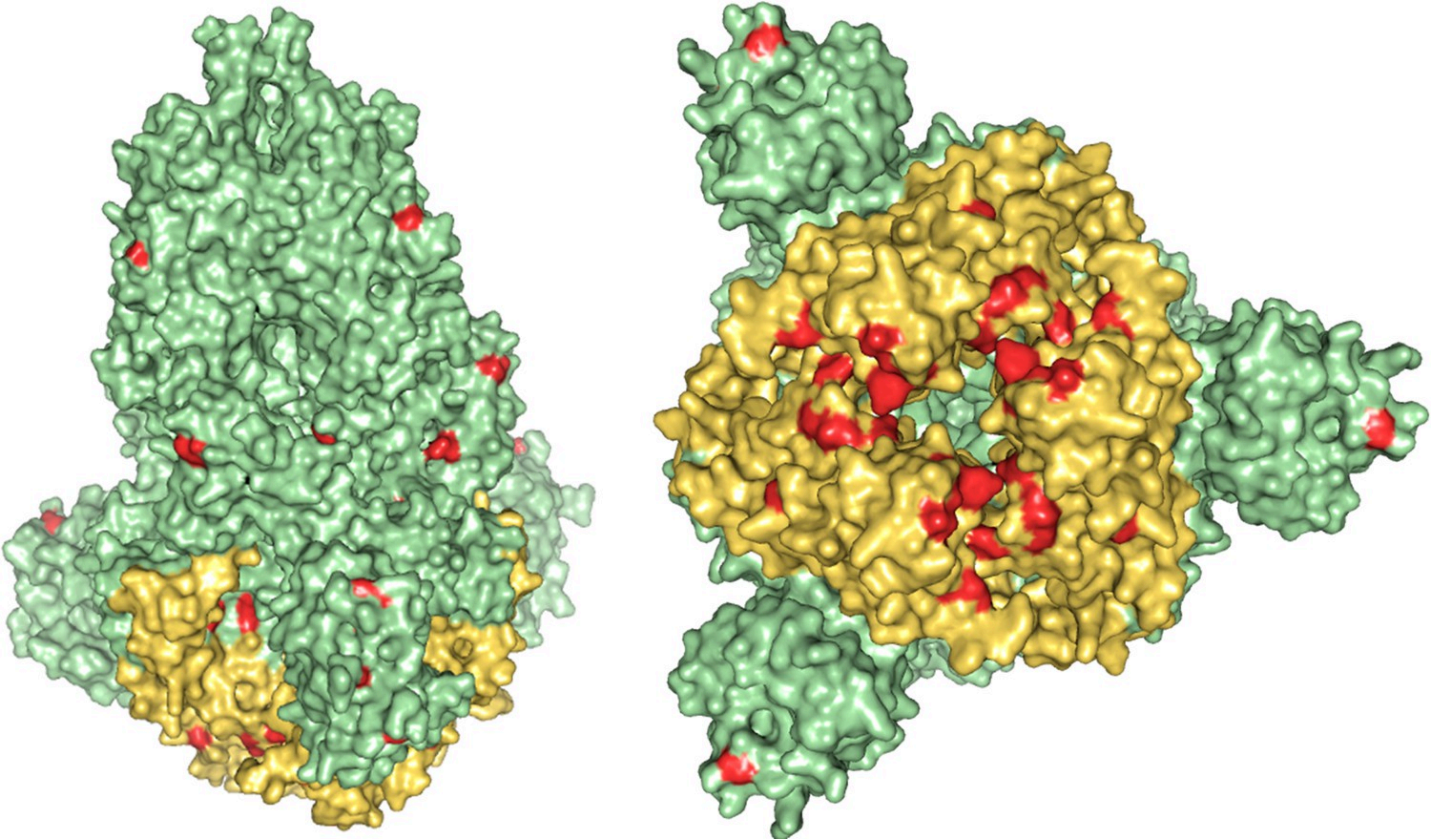
Three-dimensional structure of the Omicron variant spike protein. RBD region is colored in golden and mutations are highlighted in red. Nearly half of the mutations occur in the receptor-binding domain.

SARS-CoV-2 Omicron variant has been designated as the variant of concern due to its rapid emergence worldwide, which includes 30 mutations in the Spike protein, and nearly half of them are in the receptor-binding domain (Fig 1). Due to sequence loss, the Omicron variant has 1270 amino acids instead of the reference spike’s 1273 amino acids. It was evident from a primary analysis of the protein sequence that this variant had more Arginine, Histidine, Lysine and Glutamic acid than the reference, indicating that the spike protein is more charged (S1 Table). Furthermore, these residues were exposed to a much greater extent and contribute to binding with receptors because their pKa’s are high enough with polar side chains, which can form hydrogen bonds. On the other hand, Isoleucine and Phenylalanine were also present in higher numbers within the protein’s core, making the spike protein more hydrophobic than the reference variant. These mutations would alter its physicochemical and structural properties, which will affect the transmission rate and pathogenicity within human populations by reducing antibody-mediated protection [64].

### Effects on physicochemical properties

**Fig 2:**
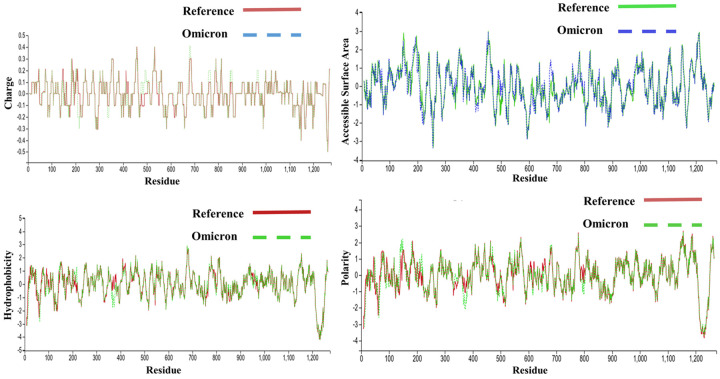
Physicochemical properties of reference variant and Omicron variant spike proteins at the residue level. In every parameter, the receptor binding domain region demonstrated larger fluctuations. **A.** Variations in electrostatic potential **B.** Differences in hydrophobicity, **C.** Variations in the accessible surface area, **D.** The difference in polarity.

**Fig 3:**
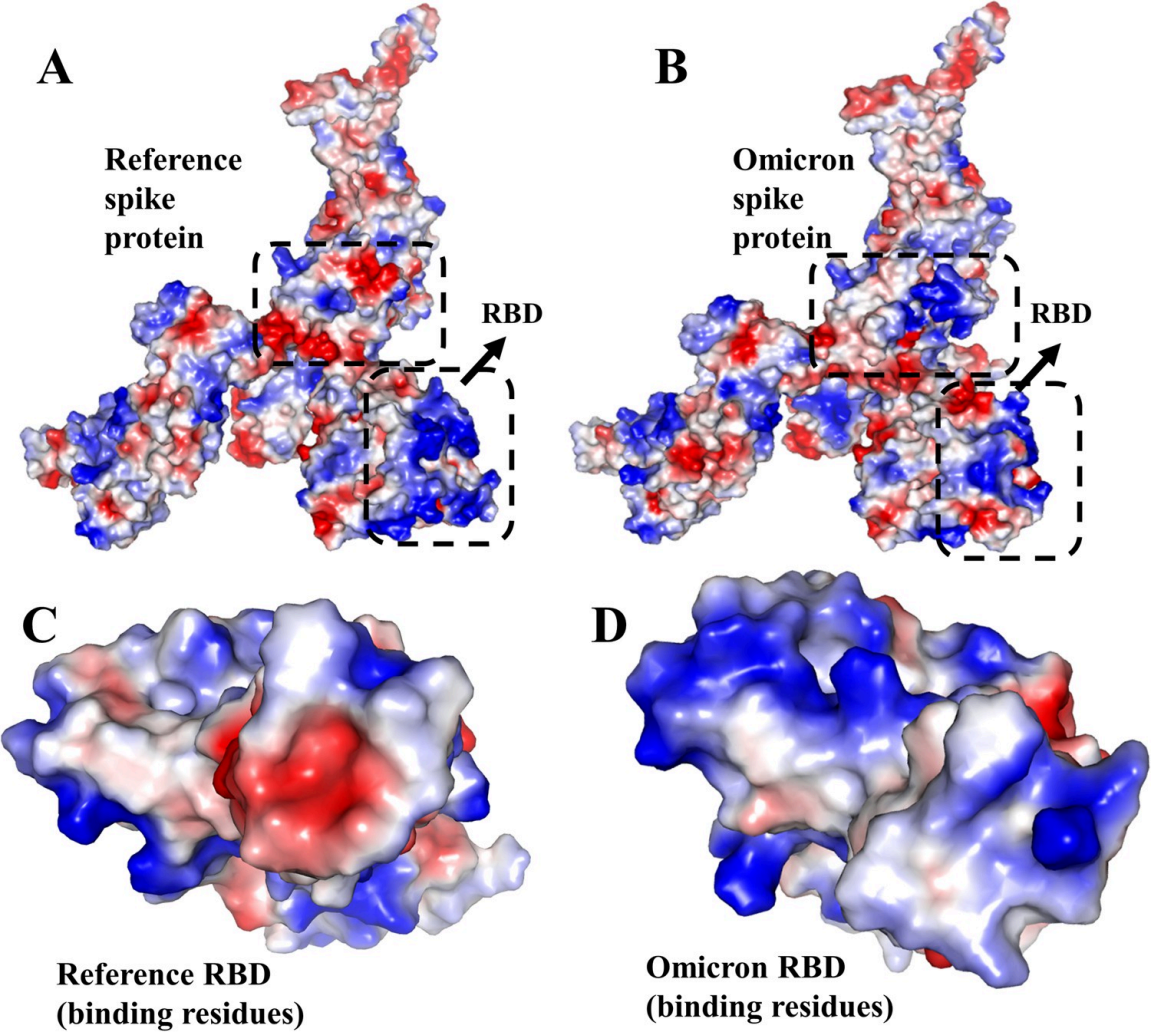
The electrostatic surface of the spike protein. The red and blue colors denote negative and positive potential respectively. Due to mutations at residues near the receptor recognition site, their electrostatic potential shifts from negative to positive, predicted to facilitate the binding with hACE2. **A.** Electrostatic potential map for the whole reference spike protein, **B.** Overall electrostatic potential map of the whole Omicron variant spike protein, **C.** High electronegativity of the recognition site at RBD of reference variant, **D.** Low electronegativity of the recognition site at RBD of Omicron variant.

Despite having three fewer amino acids, the molecular weight of the Omicron variant (141328.11 Da) was higher than the reference variant of (141178.47 Da), and the mutations were biased toward the nonpolar amino acids (Fig 2A); therefore, the hydrophobicity of the spike protein of the Omicron variant increased (Table 1 and Fig 2B). While the number of hydrophobic residues increased, the GRAVY (Grand Average of Hydrophilicity) value indicates the protein has become slightly more hydrophilic intrinsically, which is indicative of the effects of mutations on the surface accessibility of the protein (Fig 2C) due to alteration of the secondary and tertiary structural properties. Furthermore, Table 1 shows an increment of both acidic and basic residues in this variant; however, the increase in basic residues is higher (Fig 2D), resulting in a net charge of 8, which is likely to facilitate the interaction with hACE2. There was less electronegativity observed among the residues closest to the recognition of the receptor-binding domain of spike protein where the ACE-2 receptor binds (Fig 3C and 3D). In contrast, a high level of electronegativity was evident among the other residues of the domain and the complete protein of the Omicron variant (Fig 3A and 3B).

**Table 1:** Differences in the physicochemical properties of reference and Omicron variant spike proteins.

Variant	Molecular weight	Polarity		GRAVY	Charged Residues		Net Charge	Isoelectric Point	Extinction coefficient (1mg/ml)	Instability index	Aliphatic index
		Polar	Non-polar		Acidic	Basic					
Reference	141178.5	45.25%	54.75%	-0.079	8.64%	9.42%	1.5	6.62	1.037	33.01	84.67
Omicron	141314.04	45.12%	54.88%	-0.080	8.74%	10.08%	8.0	7.18	1.036	34.57	84.95

Moreover, the Omicron variant’s isoelectric point (pI) is 7.18, meaning the protein was slightly alkaline, whereas the reference variant was acidic in nature with a pI value of 6.62. Then, another important physicochemical parameter is the extinction coefficient, which is the measure of how much light is absorbed by the polypeptides. The extinction coefficient of the Omicron variant was calculated to be 1.036, while 1.037 was that of the reference variant assuming all cysteine residues are reduced. Moreover, we found that both variants of the spike proteins were stable with scores of 33.01 and 34.57 respectively for reference and Omicron, with reference being more stable. Finally, both the reference and Omicron variants had higher aliphatic index values of 84.67 and 84.95, respectively, indicating that both variants are thermostable over a wide temperature range.

### Effect on structural properties

Mutations in the spike protein were predicted to affect its structural properties. First of all, according to the secondary structural analysis, this variant had a higher fraction of alpha-helix (23.46%) than the reference (21.52%), while the beta-strand structure was decreased (Table 2). The T470-Q474 residues of the receptor-binding domain transitioning into the alpha-helix structure would increase the RBD’s stability, making the variant more transmissible and pathogenic since hACE2 interacts with the T470-F490 loop. Overall, ten residues of beta-strands and coils were predicted to be transformed into alpha-helix, but the opposite was not observed. There were, however, fourteen beta-strand residues predicted to be transformed into random coils, while seven random coil residues may be transformed into beta-strand residues. Then, the mutations influenced the solvent accessibility of 154 residues and made the variant more hydrophilic because a higher number of residues were exposed. Among 154 residues, 61 were exposed from the buried or intermediate state, while 54 were buried (S1 Table).

**Table 2:** Differences in structural features between reference and Omicron variant spike proteins.

	Secondary Structure			Solvent accessibility			Residual flexibility			Conserved Residues	
Variant	Alpha Helix	Beta Strand	Coil	Exposed	Intermediate	Buried	Flexible	Intermediate	Rigid	Functional	Structural
Reference	21.52%	22.07%	56.40%	30.48%	9.19%	60.33%	361	588	304	321	354
Omicron	23.46%	20.55%	55.98%	31.42%	8.66%	59.92%	353	586	311	309	394

Additionally, mutations in the spike protein changed the residual flexibility and increased the rigidity of the protein, which would affect its functionality. In the reference variant, 361 residues were predicted to be flexible, but the number decreased to 353 while rigid residues increased from 304 to 311 (Table 2). While some flexible residues gained an intermediate state without becoming rigid, very few rigid residues developed a flexible state directly, and all rigid transitions occurred among residues of the intermediate state (S1 Table). In this variant, flexibility predictions showed that the transmembrane domain and heptapeptide repeats (1213–1237, 912–984 and 1163–1213 residues) of the S2 subunit are highly flexible, which could affect the viral cell fusion with host cells. Despite the mutations affecting the protein’s residual flexibility, we did not observe any significant changes in the residual disorder of the protein implying several algorithms. Finally, the Omicron variant of the spike protein has 703 conserved residues for structure and function, compared to 675 residues in the reference variant (Table 2 and S1 Table). It was found that the structural residues of this variant increased from 354 to 394, which would likely increase the stability of the protein. There was, however, a decrease from 321 to 309 functional residues, which might have an impact on the viral fatality.

**Fig 4:**
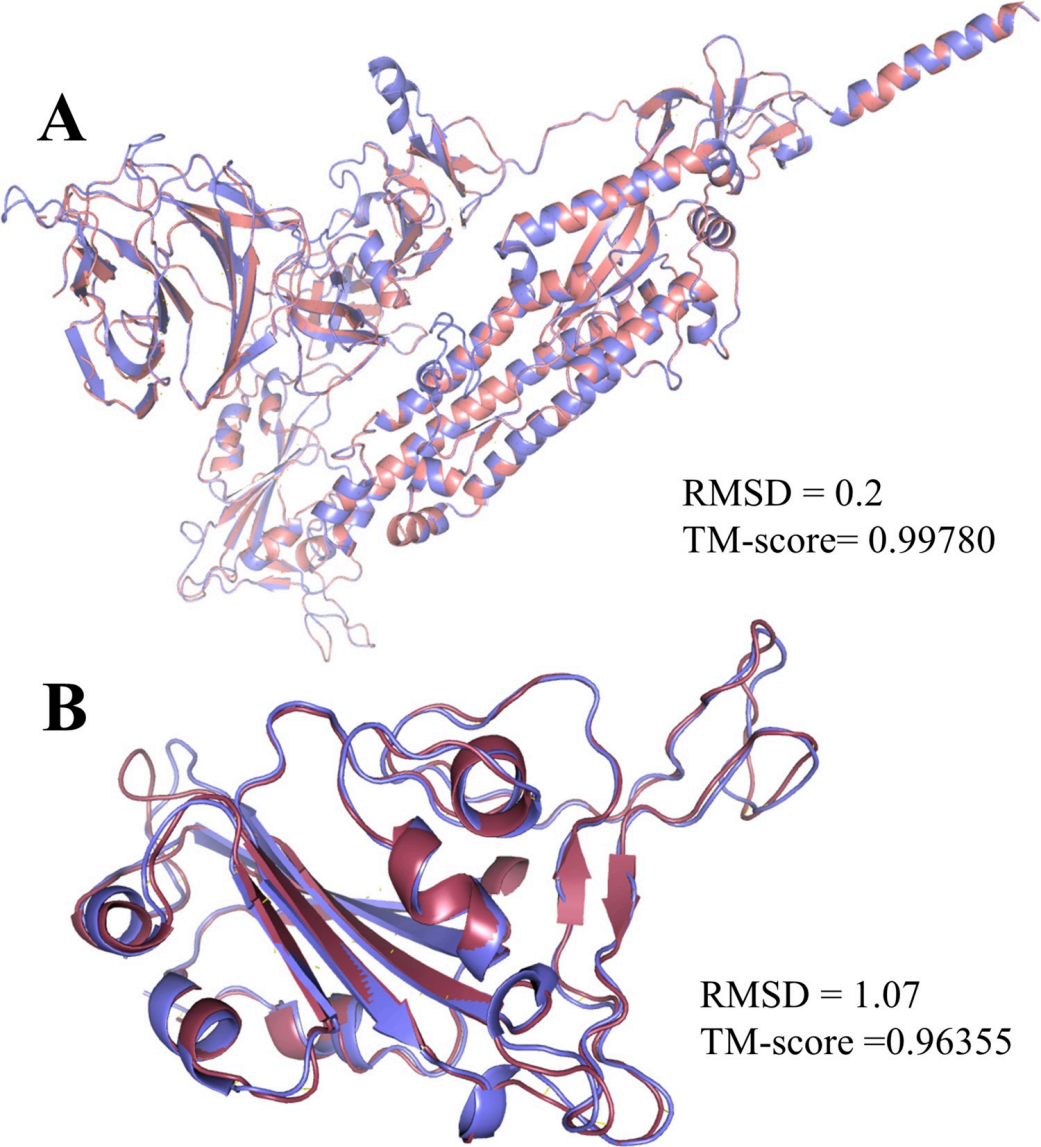
Comparison of the spike protein and the RBD of the reference and Omicron variants. In superimposing representative structures, no major changes are seen in the whole spike protein, but in the RBD, there is a difference in atomic coordinates, which would affect its interaction with receptor hACE2. **A.** Superimposed structure of the whole spike protein of the reference and the Omicron variant, **B.** Superimposed receptor-binding domain structure of the reference and the Omicron variant.

In addition, both protein structure and conformation dynamics are associated with biological functions, so we analyzed the reference and the Omicron variant’s tertiary structure further to find the structural variations. We observed an RMSD of 0.20 and a TM-score of 0.99780 (Fig 3A) between the spike proteins, indicating a higher degree of structural similarity. In contrast, a contact map overlapping analysis yielded 90.5% common contacts, with the reference variant having 89 unique contacts among the residues, whereas the Omicron variant had 438 (Fig 4A). The contact map analysis indicated some differences among the functional residues of the protein despite the RMSD value and TM-score indicating no major structural changes caused by the mutations.

The superimposition of the receptor-binding domain structures yielded an RMSD value of 1.07, indicating higher structural differences in atomic coordinates than the reference variant; however, the TM-score of 0.96355 suggests no major structural differences occurred (Fig 3B). A total of 91.3% of residue contacts were common between the reference and Omicron variant, with 50 and 34 unique contacts respectively (Fig 4B), which designated differences in the functional residues of the RBD that may affect viral transmissibility.

### Effect on stability, functionality and disease propensity

Using a combination of deep learning neural network algorithms and structure-based predictions, the effect of the mutations on the spike protein stability was predicted. There was a decrease in structural stability for all amino acid changes, but the S142H, N764K and P681H are predicted to have a significant impact (Table 3). The functional effect analysis revealed that the E484A, Y505H, T547K, N764K, N856K, and N969K mutations impair the spike protein’s function, and the rest are neutral (Table 3). One of the five mutations, E484A, is located in the receptor-binding domain, so this mutation would likely influence viral transmission. The rest of the mutations in the RBD were predicted not to affect protein function but to reduce its structural stability, which could affect it either way.

**Table 3:** The effects of mutations on the stability, functionality, and disease-proneness of the Omicron variant.

Reference AA	Position	Variant AA	Effect		
			Stability	Functionality	Disease propensity
A	67	V	Decrease Stability	Neutral	Less disease prone
T	95	I	Decrease Stability	Neutral	Less disease prone
G	142	D	Decrease Stability	Neutral	More disease prone
L	212	I	Decrease Stability	Neutral	Less disease prone
G	339	D	Decrease Stability	Neutral	More disease prone
S	371	L	Decrease Stability	Neutral	More disease prone
S	373	P	Decrease Stability	Neutral	More disease prone
S	375	F	Decrease Stability	Neutral	Less disease prone
K	417	N	Decrease Stability	Neutral	Less disease prone
G	446	S	Decrease Stability	Neutral	More disease prone
S	477	N	Decrease Stability	Neutral	Less disease prone
T	478	K	Decrease Stability	Neutral	More disease prone
E	484	A	Decrease Stability	Affect	Less disease prone
Q	493	R	Decrease Stability	Neutral	Less disease prone
G	496	S	Decrease Stability	Neutral	More disease prone
Q	498	R	Decrease Stability	Neutral	Less disease prone
N	501	Y	Decrease Stability	Neutral	More disease prone
Y	505	H	Decrease Stability	Affect	More disease prone
T	547	K	Decrease Stability	Affect	More disease prone
D	614	G	Decrease Stability	Neutral	Less disease prone
H	655	Y	Decrease Stability	Neutral	Less disease prone
N	679	K	Decrease Stability	Neutral	More disease prone
P	681	H	Decrease Stability	Neutral	Less disease prone
N	764	K	Decrease Stability	Affect	More disease prone
D	796	Y	Decrease Stability	Neutral	More disease prone
N	856	K	Decrease Stability	Affect	More disease prone
Q	954	H	Decrease Stability	Neutral	Less disease prone
N	969	K	Decrease Stability	Affect	More disease prone
L	981	F	Decrease Stability	Neutral	Less disease prone

Additionally, sixteen mutations were predicted to increase disease propensity and thirteen to decrease it. Together with the other twelve mutations, it was predicted that the E484A mutation would decrease the probability of diseases induced by the protein, which was predicted to affect the protein function. On the other hand, the other four protein function impairing mutations are predicted to increase the likelihood of disease (Table 3).

### Effects on immunologic properties

A series of analyses using conventional and neural network-based tools revealed changes in antigenicity and immunogenicity of the spike protein, but no significant changes in both pro-inflammatory and anti-inflammatory properties. In general, we found that the overall antigenicity and immunogenicity of the spike protein was increased, however in-depth studies were conducted on each prospective epitope to get better ideas. To begin with, 29 of the 133 epitopes predicted in the reference spike proteins against B-cells found to be altered in the strain Omicron. In addition, five new epitopes were observed, while six were lacking due to the higher number of mutations compared to the reference strain. Meanwhile, eight mutations were associated with an increase in antigenicity where nine mutations resulted in a significant loss of it. Using S375F as an example, the antigenicity score of the SVLYNSASFSTFKCYG epitope increased from 0.1864 to 0.8321 while T547K decreased the antigenicity score of TGTGVLTESNKKFLPF from 0.9925 to 0.5818. On the other hand, S375F reduced immunogenicity by 25% but T547K did not have any effect on the respective epitopes while, S375F increased pro-inflammatory activity by 3%, and T547K decreased it by 4%. In the case of S375F, the anti-inflammatory properties of the epitope would be increased by 10%, where, T547K was predicted to have no effect. Overall, compared to mutations altering antigenicity properties, there were relatively few mutations that could affect immunogenicity, pro-inflammatory or anti-inflammatory properties of the B-cell epitopes (S2 Table). When it came to epitopes for CD4+ T-cells, 49 of 253 were affected by the mutations where majority of them were predicted to increase the antigenicity of epitopes, while few were predicted to have negative impacts. The increase in antigenicity, however, often had the adverse effect of decreasing immunogenicity, anti-inflammatory and pro-inflammatory properties of the epitopes. As an example, the S371L mutations increased the antigenicity of the epitope YSVLYNSASFSTFKC by 69% while decreasing the immunogenicity and anti-inflammatory potential of the epitope by 74% and 7% respectively relative to the reference spike protein. While the S371L, S373P, and S375F mutations have previously been found to cause immune evasion, they were predicted to decrease immunogenicity by 117% (Table 4). However, unlike B-cell epitopes, the immunogenicity of several CD4 T-cell epitopes were predicted to be highly increased by the mutations but high level of fluctuation was not observed in inflammatory properties. For instance, T547K and G339D both increased the immunogenicity of their respective epitopes by 165% and 127%, but the pro-inflammatory potential decreased by only 2% and 3% while the anti-inflammatory potential declined by 4% and 7% respectively.

**Table 4:** Epitopes with maximum increased or decreased immunologic potentials due to mutations. Several of the mutations are in the RBD region.

Immune system	Criteria	Epitope	Mutation	Immunologic potential change
B-cell	Max^.+^ Antigenicity	FSTFKCYGVSPTKLND	S375F	107%
	Max.^-^ Antigenicity	TGTGVLTESNKKFLPF	T547K	-41%
	Max^.+^ immunogenicity	TGCVIAWNSNNLDSKV	G446S	20%
	Max.^-^ immunogenicity	NGVGYQPYRVVVLSFE	N501Y, Y505H	-32%
	Max.^+^ Pro-inflammation	LQSYGFQPTNGVGYQP	Q493R, G496S, Q498R, N501Y, Y505H	8%
	Max.^-^ Pro-inflammation	CNDPFLGVYYHKNNKS	G142D	-8%
	Max.^+^ Anti-inflammation	FSTFKCYGVSPTKLND	S375F	10%
	Max.^-^ Anti-inflammation	CNDPFLGVYYHKNNKS	G142D	-7%
CD4+ T-cell	Max.^+^ Antigenicity	SVLYNSASFSTFKCY	S371L	69%
	Max^.-^ Antigenicity	DFGGFNFSQILPDPS	D796Y	-24%
	Max^.+^ immunogenicity	CPFGEVFNATRFASV	G339D	165%
	Max^.-^ immunogenicity	DYSVLYNSASFSTFK	S371L, S373P, S375F	-117%
	Max.^+^ Pro-inflammation	PIKDFGGFNFSQILP	D796Y	6%
	Max^.-^ Pro-inflammation	SIVRFPNITNLCPFG	G339D	-6%
	Max^.+^ Anti-inflammation	LLLQYGSFCTQLNRA	N764K	4%
	Max^.-^ Anti-inflammation	YSVLYNSASFSTFKC	S371L, S373P, S375F	-7%
CD8+ T-cell	Max.^+^ Antigenicity	NSASFSTFK	N679K	121%
	Max^.-^ Antigenicity	IYKTPPIKDF	D796Y	-38%
	Max^.+^ immunogenicity	LYNSASFSTF	S371L, S373P, S375F	54%
	Max.^-^ immunogenicity	AQKFNGLTVL	N856K	-20%
	Max.^+^ Pro-inflammation	FQPTNGVGY	Q498R, N501Y, Y505H	11%
	Max.^-^ Pro-inflammation	LVRDLPQGF	L212I	-10%
	Max.^+^ Anti-inflammation	GVYFASTEK	T95I	12%
	Max.^-^ Anti-inflammation	FQPTNGVGY	Q498R, N501Y, Y505H	-11%

*Max*.^*+*^: *Maximum increased; Max*.^*-*^: *maximum decreased*.

The utmost pro-inflammation enhancing mutation for any epitope was D796Y, resulting in an increase of only 6% in epitope PIKDFGGFNFSQILP over the reference variant while N764K increased the maximum anti-inflammatory potential by 4% in the epitope LLLQYGSFCTQLNRA (Table 4). Finally, only 30 of the 259 epitopes targeting CD8+ T-cells exhibited altered antigenic, immunogenic, inflammatory properties due to mutations. Sixteen of these epitopes showed an increase in antigenicity and thirteen suggested a reduction. Two mutations S373P and S375F in the epitope NSASFSTFK enhanced immunogenicity, antigenicity and pro-inflammatory properties by 42%, 121%, and 11% respectively while the anti-inflammatory potential was anticipated to be reduced by 11%. In addition to enhancing antigenicity and immunogenicity, these mutations improved the pro-inflammatory properties of the epitope, a phenomenon that was not seen in other epitopes for any mutation therefore, this epitope needs further study. As a whole, a smaller number of mutations were associated with increasing the immunogenicity and inflammatory properties than antigenicity against CD8 T-cell epitopes. Additionally, eight of the perspective epitopes predicted from the reference sequence lacked in the Omicron variant while, nine new ones emerged. Our analysis showed that the missing epitopes in Omicron had significantly higher immunogenicity, antigenicity, and inflammatory properties than the newly appeared epitopes. The S2 Table contains details of epitopes and the effects of mutations on them.

### Effect on binding interaction with hACE2

Infectivity, transmission, and pathogenesis are largely determined by the binding affinity of the virus towards the receptor, so mutations in the receptor-binding domain of the virus can greatly influence its activities. Since SARS-CoV-2 interacts with hACE2 through its C-terminal domain, mutations at key residues would affect the interaction with hACE2. In this part of the study, we found that the binding affinity of the Omicron variant differs from the reference variant. Our results from the HDOCK server showed that the docking score and binding affinity for the Omicron variant were -343.56 and -11.8 kcal/mol, compared to -310.19 and -11.5 kcal/mol for the reference variant (Table 5). Moreover, the ClusPro server provided us with the docking score and binding affinity for Omicron variants -703.8 and -14.7, respectively, compared to -639.3 and -11.9 for the reference variant (Table 5). Thus, it was evident that the Omicron variant exhibits a stronger binding affinity to the hACE2 than the reference variant, implying a potential for higher viral transmission.

**Table 5:** Differences in interfacial contacts between reference and Omicron variant with hACE2.

Interfacial contacts		Reference variant	Omicron Variant
Binding affinity (kcal/mol)	1. ClusPro	-11.9	-14.7
	2. HDOCK	-11.5	-11.8
Hydrophobic interaction		4	6
Main chain-main chain hydrogen bonds		1	0
Main chain-side chain hydrogen bonds		5	14
Side chain-side chain hydrogen bonds		11	18
Ionic interactions		3	7
Aromatic-Sulphur interactions		1	0
Pi-cation interactions		0	3

**Fig 5:**
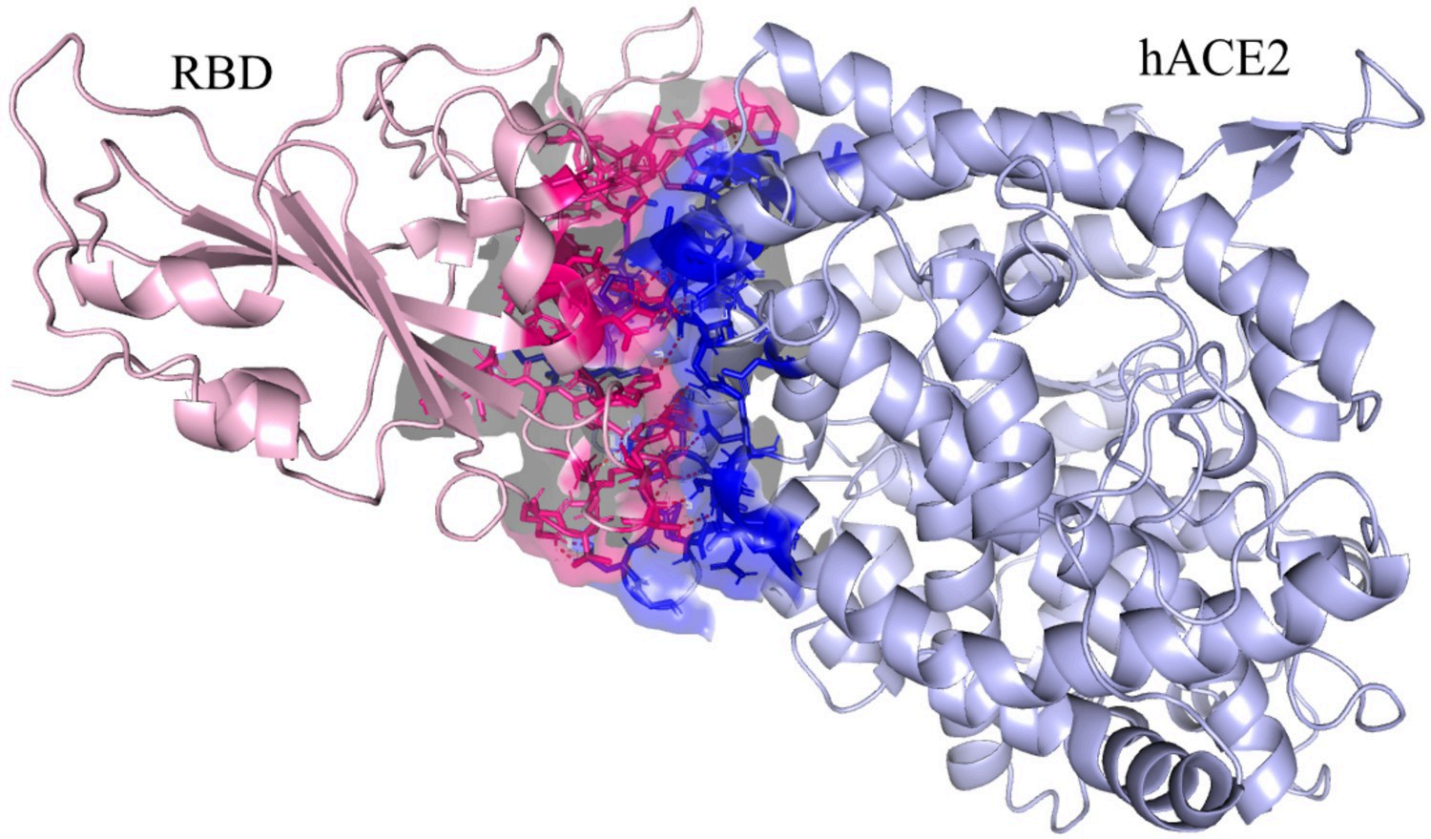
Interfacial contacts of the receptor-binding domain of the Omicron variant with hACE2.

There are significantly more interactions with hACE2 than in the reference (Fig 5), with 48 versus 25 interfacial contacts in the Omicron variant, most of which are hydrogen bonds (Table 5). Our study of protein-protein interaction in the reference variant found four hydrophobic interactions involving Y489 and F486 residues. These interactions were not present in the Omicron variant; however, six new hydrophobic bonds involving Y446, K452, I469, A481 and F487 were discovered. Pi-Cation interactions were observed at three new sites with Y446, Y486 and Y498 residues in the Omicron variant, but there were no Pi-Cation interactions in the reference variant, while the only aromatic bond of the reference was absent in Omicron. Additionally, the number of ionic interactions increased from three to seven when the RBDs of Omicron spikes bind with hACE2. However, the maximum discrepancy was observed regarding hydrogen bond formation, where the only hydrogen bond between the main chains of the reference RBD and hACE2 was absent from the Omicron variant. Meanwhile, the number of bonds formed between the main chain and side chain increased dramatically from 5 to 14, and the number of bonds formed between side chains rose from 11 to 18.

Six of the fifteen mutations found in the RBD of the spike protein in the Omicron variant directly affected its interaction with hACE2. For instance, E484 was in ionic contact with K31 in the reference structure, but the Omicron variant (E484A) lost this ionic interaction; instead, A484 formed two hydrophobic interactions with L79 and M82 residues of hCAE2. The most drastic effect on the interactions took place due to the Q498R mutation, which is located near the hACE2 recognition loop structure of RBD. There was a hydrogen bond present between the Q498 of the reference RBD and the Q42 residue of hACE2; however, the Q489R mutation impaired that bond and led to the formation of several new hydrogen bonds among the residues of the main chains and side chains, as well as ion interactions with E208 and D216 residues of hACE2 (S1 Table). In addition, the N501Y mutations, which were previously reported for the alpha variant, also increased the binding affinity for the Omicron variant because two hydrogen bonds and one Pi-Cation bond were formed instead of one hydrogen bond in the reference variant. In the supplementary file, we provide a full comparison of the protein-protein interactions of Omicron RBD and reference RBD with hACE2. To summarize, it is evident that mutations increased the binding affinity between the receptor-binding domain and hACE2, which is a key factor in the transmissibility and pathogenesis of SARS-CoV-2.

### MD simulation result on RBD-ACE2 complex

Using molecular dynamics simulation, we observed new interactions formed with hACE2 while retaining many of the old ones found in the reference sequence. These interactions were attributed to additional residues at the interface (Q474, G476, N477, K478, A484, F486, and Y489). Despite this, we found several interaction disruptions; for instance, the salt bridge between E484 and K31 was lost, the hydrogen bond between K417 and D30 was damaged, and other interactions were disrupted at residues F456, A475, and G502. According to our analysis of 15 mutations that have been reported to occur in the RBD of the Omicron variant, 6 of these mutations were found to have additional effects on the binding of the Omicron RBD with hACE2.

**Fig 6:**
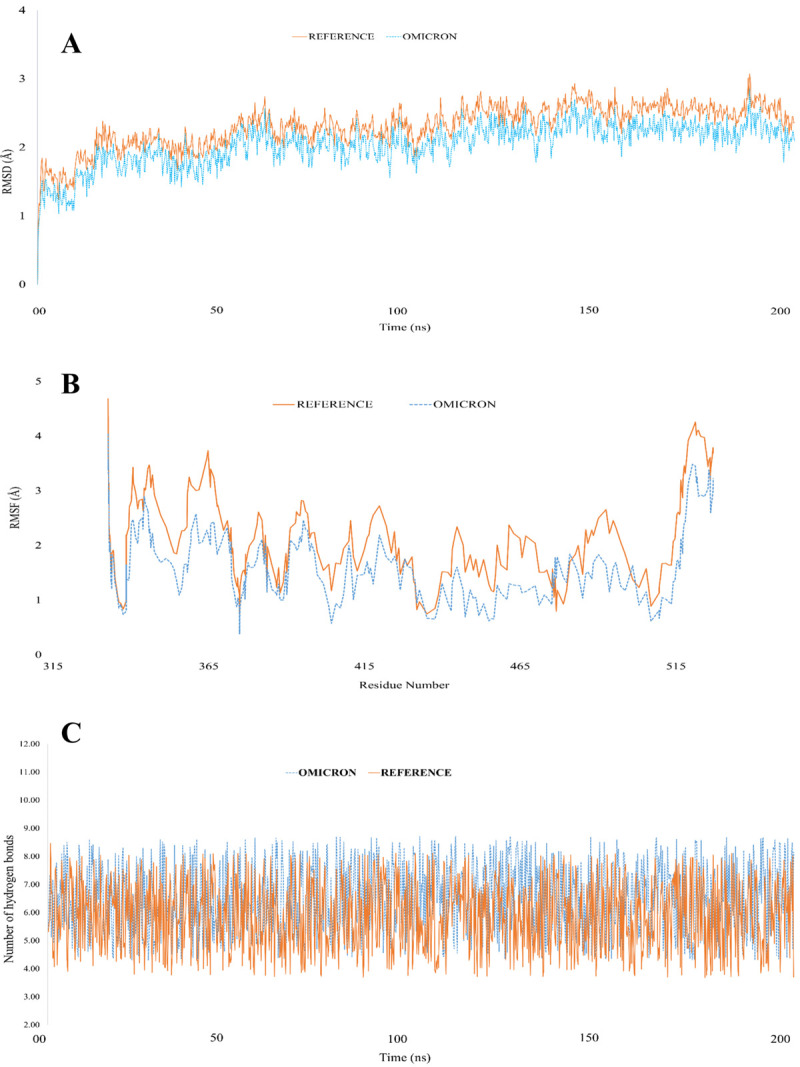
Representation of the stability and hydrogen bonds in the RBD-hACE2 complex structure in the reference and Omicron variants. **A.** Root mean square deviation (RMSD) of RBD-hACE2 complex. **B.** Root mean square fluctuations (RMSF) of the receptor binding residues in RBD-hACE2 complex. **C.** Number of hydrogen bonds of the RBD observed with hACE2 during simulations.

Furthermore, on average, we found 6.89±1.28 hydrogen bonds between Omicron RBD and hACE2, as compared to 5.52±1.26 hydrogen bonds with the reference variant (Fig 6C). This indicates that the Omicron variant enhances the interaction between RBD and hACE2 compared to the reference variant. Interestingly, mutations of Q498R and N501Y were found to form new hydrogen bonds with occupancy higher than 15%. In contrast, mutations of K417N and Y505H led to the loss of hydrogen bonds, and some mutations did not affect hydrogen bonding, for example, the K493-E35 bond resulting from the Q493K mutation. After that, we calculated the binding energies of the hACE2 complex in comparison to either the RBD of Omicron and the reference, and it was found that the binding energy of hACE2 is lower when binding to Omicron RBD (-98.02 kcal/mol) as opposed to the reference (-83.7 kcal/mol).

In addition, the root-mean-square-deviation (RMSD) for the reference ACE2-RBD complex was 2.61 Å (Fig 6A). In comparison, the value was 2.05 Å for the Omicron RBD-ACE2 complex; a finding that indicates the mutations did not lead to significantly reduced stability of RBD but instead increased the stability of the Omicron RBD-ACE2 complex over the reference variant. Additionally, we calculated the root-mean-square fluctuations (RMSF) from the trajectory data and found that the RBD of the Omicron variant is more rigid than the reference variant and that the RMSF values averaged to 1.7 Å and 2.2 Å, respectively (Fig 6B). Interestingly, the mutated residues at the interface of the hACE2 showed reduced fluctuations compared to the reference variant at the interface. Thus, when taking into account the number of hydrogen bonds, binding energies, RMSD, RMSF, and RMSD, Omicron RBD-ACE2 appears to be more stable than the reference complex.

## Discussion

The World Health Organization deems Micron a variant of concern due to its rapid transmission rate and the unusually large number of amino acid mutations in its spike protein. In this study, we investigated how the mutations affect the spike protein from physicochemical, structural, functional and immunologic standpoints and how they modulate its interactions with the host protein ACE2.

To begin with, despite a decline in overall hydrophobicity resulting from changes in surface accessibility of several residues due to mutations, the hydrophobic residues are increased in number in the spike protein of the Omicron variant, which is required for the stability of the protein and make the protein’s core [65, 66]. In addition, changes in the hydrophobicity of amino acids may alter the structure of the epitope in the receptor-binding domain of the spike protein, which is likely to affect the immune response to the virus [64, 67]. In Omicron, the number of positively charged amino acids in the spike protein has increased by nine in comparison with the reference variant. Especially, a number of changes occurred in the S1/S2 domain where a basic amino acid replaced a negatively charged amino acid, indicating a selective advantage to bind to host cells. Moreover, the electrostatic potential analysis indicated that L452R, T478K, Q493R, Q498R, and Y501H mutations increased the number of cationic residues near the receptor-binding motif, which may strongly interact with negatively charged residues in ACE2.

In addition, the structure of the spike protein of the Omicron variant consists of a higher percentage of alpha-helix structures, which are known for increasing conformational stability of proteins; therefore, it is expected that the stability of the spike protein of the Omicron variant will also be increased [68–70]. We found that T470-Q474 residues of the receptor-binding loop might undergo a coil to alpha-helix transition, which might facilitate the formation of strong binding with hACE2 and enhance transmissibility. However, no significant change was detected in the residual disorder property, despite a reduction in overall residual flexibility. Possibly, the reduction in flexibility will affect its stability and activity, as well as its ability to be recognized by current vaccines and therapies. On the other hand, the transmembrane domain of the S2 subunit of the spike protein was predicted to be more flexible, which may facilitate viral fusion with host cells [71].

Then, the Omicron variant is predicted to contain fewer conserved functional residues. Contact map overlapping analysis also supported the prediction since only 90.5% of common contacts with reference protein was observed, suggesting differences among the functional residues. The receptor-binding domain of the proteins, however, showed 91.3% common contacts with an RMSD value of 1.07, and a TM-score of 0.96355, while the respective values for the whole protein were 0.2 and 0.99780. Based on these results, it is evident that the greater part of the changes took place in the RBD region, which can also be explained by the presence of half the mutations there. The functional RBD domain is essential for recognition of and binding to ACE2, so any mutation in the RBD domain could influence its function as well as its affinity for binding to the ACE2 receptor [12, 22]. Interestingly, we observed that three mutations in the RBD region can affect its function; fourteen mutations can exacerbate the severity of the disease; and all mutations will decrease its stability, which will, in turn, affect the transmission and virulence potential of SARS-CoV-2.

Furthermore, the spike glycoprotein is exposed antigen of SARS-CoV-2, which triggers humoral as well as cell-mediated immunity therefore, most of the current vaccines and therapies target the spike RBD-ACE2 interaction. However, SARS-CoV-2 can, like other viruses, develop immune evasion strategies through antigenic variations in order to escape these immune responses [15, 72]. There have previously been several studies showing that SARS-CoV-2 variants can escape the immune system because of key mutations [73, 74]. Consequently, identifying and analyzing the effects of mutation on immunologic properties is critical for a better understanding of pathogenesis and transmission. Based on our extensive in silico analysis of large sets of epitopes, we found that most of the previously reported mutations, which helped in immune escape, would reduce antigenicity and immunogenicity. As an example, K417N mutations cause immune escape against class I antibodies, which we found to decrease humoral immunity by 7% [73]. The mutation E484A had been reported to confer resistance to multiple antibodies, and our prediction suggested that it could reduce the antigenic and immunogenic potential of the corresponding B-cell epitope by 6% and 5% respectively [75]. In addition to humoral immunity, several mutations have been predicted to decrease the immunogenic potential of T-cell epitopes. For example, S371L, S373P, and S375F mutations have previously been reported to cause immune evasion, and our analysis showed that they decreased immunogenicity by 117% of a + CD+ T-cell epitope [76]. On the other hand, the mutation K417N might decrease the immunogenic and antigenic potential of an epitope predicted to produce CD8+ T-cell mediated immunity by 20% and 18%, respectively. Despite the fact that some mutations might lower the immunogenicity of the epitopes, our analyses illustrated that most mutations tended to increase immunogenic potentials (S2 Table). In addition, we observed that the mutations had no substantial impact on the pro-inflammatory or anti-inflammatory properties of the epitopes (S2 Table). In light of this, we assume that the combination of higher immunogenicity and antigenicity as well as lower inflammatory potentials of the epitopes account for the lower disease severity.

Additionally, several mutations in the Omicron have previously been reported in other VOCs, and all of these mutations correlated with immune evasion, increased transmissibility, and stronger binding to hACE2 [8, 72, 77, 78]. Therefore, this variant is also expected to exhibit higher transmissibility and increased immune evasion. The extent of viral transmissibility is largely determined by its ability to bind to its receptor, and we found that the Omicron variant binds to hACE2 more strongly than the reference strain. T478K and N501Y mutations have been reported to enhance the interaction with hACE2 in the Delta variant, which are also present in this variant [79]. The N501, Q493, Q498 residues are crucial in RBD-hACE2 interactions since they form polar contacts with the ACE2 residues K31 and K353. Q498R mutation led to a dramatic change in the protein-protein interactions, forming several hydrogen bonds with V209, D216, E208 and Q210 residues, and ion interactions with E208 and D216 residues of hACE2. Moreover, the Omicron variant carries the D614G and P681H mutations, previously described as critical mutations that enhance the transmission and infectivity of SARS-CoV-2 [80–82]. Overall, the number of interfacial contacts in the Omicron variant is significantly increased and the majority are hydrogen bonds, which explains the increased binding affinity.

Lastly, the molecular dynamics simulations revealed that residues Q474, G476, N477, K478, A484, F486, and Y489 at the interface enhanced the interactions by forming salt bridges and hydrogen bonds while maintaining the previous interactions. Moreover, on average, we observed 6.89±1.28 and 5.52±1.26 hydrogen bonds with binding energies of -98.02 kcal/mol and -83.7 kcal/mol for the Omicron and reference variants, respectively when RBD was bound with hACE2. An increase in interactions due to the mutations of the receptor-binding domain of spike protein induced a higher binding affinity with hACE2 and stabilized the RBD-hACE2 complex. Moreover, the RMSD and RMSF values for the hACE2-RBD complex of the Omicron variant were 2.05 Å and 1.7 Å, while, 2.61 Å and 2.2 Å were for the reference variant, signifying the higher integrity of the complex. Finally, we observed that when Omicron spike protein was bound to hACE2 there were fewer fluctuations among the atoms than when reference spike protein was bound. Hence, the Omicron spike protein exhibits better binding affinity and greater structural stability to ACE2-receptor than the reference protein.

## Conclusion

In light of the large number of mutations observed in the spike protein of the SARS-CoV-2, Omicron, a new variant of the virus poses serious concerns. Therefore, in the following work, we used *in silico* computational methods to study the dynamic changes in spike protein of the SARS-CoV-2 caused by the large number of mutations. The results of our analysis revealed significant differences with respect to physicochemical, structural, functional, and immunologic changes, the enhanced binding affinity of RBD with hACE2, and a lower residual fluctuation, which might facilitate the greater transmission. The case reports about the rapid spread of this variant in different parts of the world are consistent with our observations. Therefore, this variant should be subjected to more comprehensive research.

## Supporting information

S1 Table Details on amino acid composition and effects of mutations on the physicochemical, structural, functional and binding properties of the spike protein. (XLSX)

S2 Table Detail effects of the mutations on antigenicity, immunogenicity, pro-inflammation and anti-inflammation properties of the epitopes predicted for B-cell, CD4+ T-cell and CD8+ T-cell mediated immunity. (XLSX)

## References

[1] Wu YC, Chen CS, Chan YJ. The outbreak of COVID-19: An overview. Journal of the Chinese Medical Association. 2020. pp. 217–220. doi: 10.1097/JCMA.0000000000000270 32134861 PMC7153464

[2] Ozili PK, Arun T. Spillover of COVID-19: Impact on the Global Economy. SSRN Electron J. 2020. doi: 10.2139/ssrn.3562570

[3] Mathieu E, Ritchie H, Ortiz-Ospina E, Roser M, Hasell J, Appel C, et al. A global database of COVID-19 vaccinations. Nat Hum Behav. 2021;5: 947–953. doi: 10.1038/s41562-021-01122-8 33972767

[4] Tregoning JS, Flight KE, Higham SL, Wang Z, Pierce BF. Progress of the COVID-19 vaccine effort: viruses, vaccines and variants versus efficacy, effectiveness and escape. Nature Reviews Immunology. 2021. pp. 626–636. doi: 10.1038/s41577-021-00592-1 34373623 PMC8351583

[5] Wang L, Zhou T, Zhang Y, Yang ES, Schramm CA, Shi W, et al. Ultrapotent antibodies against diverse and highly transmissible SARS-CoV-2 variants. Science (80-). 2021;373. doi: 10.1126/science.abh1766 34210892 PMC9269068

[6] World Health Organization. Classification of Omicron (B.1.1.529): SARS-CoV-2 Variant of Concern. https://www.who.int/news/item/26-11-2021-classification-of-omicron-(b.1.1.529)-sars-cov-2-variant-of-concern. In: Classification of Omicron (B.1.1.529): SARS-CoV-2 Variant of Concern. https://www.who.int/news/item/26-11-2021-classification-of-omicron-(b.1.1.529)-sars-cov-2-variant-of-concern [Internet]. 2021 p. 1. Available: https://www.who.int/news/item/26-11-2021-classification-of-omicron-(b.1.1.529)-sars-cov-2-variant-of-concern.

[7] Dyer O. Covid-19: South Africa’s surge in cases deepens alarm over omicron variant. BMJ. 2021;375: n3013. doi: 10.1136/bmj.n3013 34862184

[8] Choi JY, Smith DM. SARS-CoV-2 variants of concern. Yonsei Medical Journal. 2021. pp. 961–968. doi: 10.3349/ymj.2021.62.11.961 34672129 PMC8542474

[9] Karim SSA, Karim QA. Omicron SARS-CoV-2 variant: a new chapter in the COVID-19 pandemic. Lancet. 2021;398: 2126–2128. doi: 10.1016/S0140-6736(21)02758-6 34871545 PMC8640673

[10] Callaway E. Heavily mutated Omicron variant puts scientists on alert. Nature. 2021;600: 21. doi: 10.1038/d41586-021-03552-w 34824381

[11] Li S, Li S, Disoma C, Zheng R, Zhou M, Razzaq A, et al. SARS-CoV-2: Mechanism of infection and emerging technologies for future prospects. Reviews in Medical Virology. 2021. doi: 10.1002/rmv.2168 35349206

[12] Scialo F, Daniele A, Amato F, Pastore L, Matera MG, Cazzola M, et al. ACE2: The Major Cell Entry Receptor for SARS-CoV-2. Lung. 2020. pp. 867–877. doi: 10.1007/s00408-020-00408-4 33170317 PMC7653219

[13] Ng KT, Mohd-Ismail NK, Tan YJ. Spike s2 subunit: The dark horse in the race for prophylactic and therapeutic interventions against sars-cov-2. Vaccines. 2021. pp. 1–12. doi: 10.3390/vaccines9020178 33672450 PMC7923282

[14] Shah P, Canziani GA, Carter EP, Chaiken I. The Case for S2: The Potential Benefits of the S2 Subunit of the SARS-CoV-2 Spike Protein as an Immunogen in Fighting the COVID-19 Pandemic. Front Immunol. 2021;12. doi: 10.3389/fimmu.2021.637651 33767706 PMC7985173

[15] Harvey WT, Carabelli AM, Jackson B, Gupta RK, Thomson EC, Harrison EM, et al. SARS-CoV-2 variants, spike mutations and immune escape. Nature Reviews Microbiology. 2021. pp. 409–424. doi: 10.1038/s41579-021-00573-0 34075212 PMC8167834

[16] Prévost J, Finzi A. The great escape? SARS-CoV-2 variants evading neutralizing responses. Cell Host Microbe. 2021;29: 322–324. doi: 10.1016/j.chom.2021.02.010 33705702 PMC7945862

[17] Rodriguez-Coira J, Sokolowska M. SARS-CoV-2 candidate vaccines—composition, mechanisms of action and stages of clinical development. Allergy Eur J Allergy Clin Immunol. 2021;76: 1922–1924. doi: 10.1111/all.14714 33340417

[18] Mascellino MT, Di Timoteo F, De Angelis M, Oliva A. Overview of the main anti-sars-cov-2 vaccines: Mechanism of action, efficacy and safety. Infection and Drug Resistance. 2021. pp. 3459–3476. doi: 10.2147/IDR.S315727 34511939 PMC8418359

[19] Brouwer PJM, Caniels TG, van der Straten K, Snitselaar JL, Aldon Y, Bangaru S, et al. Potent neutralizing antibodies from COVID-19 patients define multiple targets of vulnerability. Science (80-). 2020;369: 643–650. doi: 10.1126/science.abc5902 32540902 PMC7299281

[20] Wang K, Chen W, Zhang Z, Deng Y, Lian JQ, Du P, et al. CD147-spike protein is a novel route for SARS-CoV-2 infection to host cells. Signal Transduct Target Ther. 2020;5. doi: 10.1038/s41392-020-00426-x 33277466 PMC7714896

[21] Cantuti-Castelvetri L, Ojha R, Pedro LD, Djannatian M, Franz J, Kuivanen S, et al. Neuropilin-1 facilitates SARS-CoV-2 cell entry and infectivity. Science (80-). 2020;370. doi: 10.1126/science.abd2985 33082293 PMC7857391

[22] Clausen TM, Sandoval DR, Spliid CB, Pihl J, Perrett HR, Painter CD, et al. SARS-CoV-2 Infection Depends on Cellular Heparan Sulfate and ACE2. Cell. 2020;183: 1043–1057.e15. doi: 10.1016/j.cell.2020.09.033 32970989 PMC7489987

[23] Mallapaty S. Researchers fear growing COVID vaccine hesitancy in developing nations. Nature. 2021;601(7892):174–175. 10.1038/d41586-021-03830-7 34949861

[24] Lupala C, Ye Y, Chen H, Su X, Liu H. Mutations on RBD of SARS-CoV-2 Omicron variant result in stronger binding to human ACE2 receptor. Biochemical and Biophysical Research Communications. 2022;590:34–41. doi: 10.1016/j.bbrc.2021.12.079 34968782 PMC8702632

[25] Rath S, Padhi A, Mandal N. Scanning the RBD-ACE2 molecular interactions in Omicron variant. Biochemical and Biophysical Research Communications. 2022;592:18–23. doi: 10.1016/j.bbrc.2022.01.006 35007846 PMC8732900

[26] GISAID. GISAID Initiative. Adv Virus Res. 2020;2008: 1–7.

[27] Bateman A, Martin MJ, Orchard S, Magrane M, Agivetova R, Ahmad S, et al. UniProt: The universal protein knowledgebase in 2021. Nucleic Acids Res. 2021;49: D480–D489. doi: 10.1093/nar/gkaa1100 33237286 PMC7778908

[28] Liu B, Liu K, Zhang H, Zhang L, Bian Y, Huang L. CoV-Seq, a new tool for SARS-CoV-2 genome analysis and visualization: Development and usability study. J Med Internet Res. 2020;22. doi: 10.2196/22299 32931441 PMC7537720

[29] Rice P, Longden L, Bleasby A. EMBOSS: The European Molecular Biology Open Software Suite. Trends in Genetics. 2000. pp. 276–277. doi: 10.1016/s0168-9525(00)02024-2 10827456

[30] Sievers F, Higgins DG. Clustal Omega for making accurate alignments of many protein sequences. Protein Sci. 2018;27: 135–145. doi: 10.1002/pro.3290 28884485 PMC5734385

[31] Burley SK, Berman HM, Bhikadiya C, Bi C, Chen L, Di Costanzo L, et al. RCSB Protein Data Bank: Biological macromolecular structures enabling research and education in fundamental biology, biomedicine, biotechnology and energy. Nucleic Acids Res. 2019;47: D464–D474. doi: 10.1093/nar/gky1004 30357411 PMC6324064

[32] Artimo P, Jonnalagedda M, Arnold K, Baratin D, Csardi G, De Castro E, et al. ExPASy: SIB bioinformatics resource portal. Nucleic Acids Res. 2012;40. doi: 10.1093/nar/gks400 22661580 PMC3394269

[33] Bonnal RJP, Aerts J, Githinji G, Goto N, Maclean D, Miller CA, et al. Biogem: An effective tool-based approach for scaling up open source software development in bioinformatics. Bioinformatics. 2012;28: 1035–1037. doi: 10.1093/bioinformatics/bts080 22332238 PMC3315718

[34] Bartonek L, Zagrovic B. VOLPES: an interactive web-based tool for visualizing and comparing physicochemical properties of biological sequences. Nucleic Acids Res. 2019;47: W632–W635. doi: 10.1093/nar/gkz407 31114895 PMC6602475

[35] Drozdetskiy A, Cole C, Procter J, Barton GJ. JPred4: A protein secondary structure prediction server. Nucleic Acids Res. 2015;43: W389–W394. doi: 10.1093/nar/gkv332 25883141 PMC4489285

[36] Klausen MS, Jespersen MC, Nielsen H, Jensen KK, Jurtz VI, Sønderby CK, et al. NetSurfP-2.0: Improved prediction of protein structural features by integrated deep learning. Proteins Struct Funct Bioinforma. 2019;87: 520–527. doi: 10.1002/prot.25674 30785653

[37] Hu G, Katuwawala A, Wang K, Wu Z, Ghadermarzi S, Gao J, et al. flDPnn: Accurate intrinsic disorder prediction with putative propensities of disorder functions. Nat Commun. 2021;12. doi: 10.1038/s41467-021-24773-7 34290238 PMC8295265

[38] De Brevern AG, Bornot A, Craveur P, Etchebest C, Gelly JC. PredyFlexy: Flexibility and local structure prediction from sequence. Nucleic Acids Res. 2012;40. doi: 10.1093/nar/gks482 22689641 PMC3394303

[39] Ashkenazy H, Abadi S, Martz E, Chay O, Mayrose I, Pupko T, et al. ConSurf 2016: an improved methodology to estimate and visualize evolutionary conservation in macromolecules. Nucleic Acids Res. 2016;44: W344–W350. doi: 10.1093/nar/gkw408 27166375 PMC4987940

[40] Bernhofer M, Dallago C, Karl T, Satagopam V, Heinzinger M, Littmann M, et al. PredictProtein—Predicting protein structure and function for 29 years. Nucleic Acids Res. 2021;49: W535–W540. doi: 10.1093/nar/gkab354 33999203 PMC8265159

[41] Jumper J, Evans R, Pritzel A, Green T, Figurnov M, Ronneberger O, et al. Highly accurate protein structure prediction with AlphaFold. Nature. 2021;596: 583–589. doi: 10.1038/s41586-021-03819-2 34265844 PMC8371605

[42] Zhang Y, Skolnick J. TM-align: A protein structure alignment algorithm based on the TM-score. Nucleic Acids Res. 2005;33: 2302–2309. doi: 10.1093/nar/gki524 15849316 PMC1084323

[43] Vehlow C, Stehr H, Winkelmann M, Duarte JM, Petzold L, Dinse J, et al. CMView: Interactive contact map visualization and analysis. Bioinformatics. 2011;27: 1573–1574. doi: 10.1093/bioinformatics/btr163 21471016

[44] Cao H, Wang J, He L, Qi Y, Zhang JZ. DeepDDG: Predicting the Stability Change of Protein Point Mutations Using Neural Networks. J Chem Inf Model. 2019;59: 1508–1514. doi: 10.1021/acs.jcim.8b00697 30759982

[45] Rodrigues CHM, Pires DEV, Ascher DB. DynaMut2: Assessing changes in stability and flexibility upon single and multiple point missense mutations. Protein Sci. 2021;30: 60–69. doi: 10.1002/pro.3942 32881105 PMC7737773

[46] Choi Y, Chan AP. PROVEAN web server: A tool to predict the functional effect of amino acid substitutions and indels. Bioinformatics. 2015;31: 2745–2747. doi: 10.1093/bioinformatics/btv195 25851949 PMC4528627

[47] Sim NL, Kumar P, Hu J, Henikoff S, Schneider G, Ng PC. SIFT web server: Predicting effects of amino acid substitutions on proteins. Nucleic Acids Res. 2012;40. doi: 10.1093/nar/gks539 22689647 PMC3394338

[48] Laskowski RA, Stephenson JD, Sillitoe I, Orengo CA, Thornton JM. VarSite: Disease variants and protein structure. Protein Sci. 2020;29: 111–119. doi: 10.1002/pro.3746 31606900 PMC6933866

[49] Reynisson B, Alvarez B, Paul S, Peters B, Nielsen M. NetMHCpan-4.1 and NetMHCIIpan-4.0: Improved predictions of MHC antigen presentation by concurrent motif deconvolution and integration of MS MHC eluted ligand data. Nucleic Acids Res. 2021;48: W449–W454. doi: 10.1093/NAR/GKAA379 32406916 PMC7319546

[50] Dhanda SK, Mahajan S, Paul S, Yan Z, Kim H, Jespersen MC, et al. IEDB-AR: immune epitope database—analysis resource in 2019. Nucleic Acids Res. 2019;47: W502–W506. doi: 10.1093/nar/gkz452 31114900 PMC6602498

[51] Manavalan B, Govindaraj RG, Shin TH, Kim MO, Lee G. iBCE-EL: A New Ensemble Learning Framework for Improved Linear B-Cell Epitope Prediction. Front Immunol. 2018;9. doi: 10.3389/fimmu.2018.01695 30100904 PMC6072840

[52] Doytchinova IA, Flower DR. VaxiJen: A server for prediction of protective antigens, tumour antigens and subunit vaccines. BMC Bioinformatics. 2007;8. doi: 10.1186/1471-2105-8-4 17207271 PMC1780059

[53] Manavalan B, Shin TH, Kim MO, Lee G. PIP-EL: A New Ensemble Learning Method for Improved Proinflammatory Peptide Predictions. Front Immunol. 2018;9. doi: 10.3389/fimmu.2018.01783 30108593 PMC6079197

[54] Manavalan B, Shin TH, Kim MO, Lee G. AIPpred: Sequence-based prediction of anti-inflammatory peptides using random forest. Front Pharmacol. 2018;9. doi: 10.3389/fphar.2018.00276 29636690 PMC5881105

[55] Kozakov D, Hall DR, Xia B, Porter KA, Padhorny D, Yueh C, et al. The ClusPro web server for protein-protein docking. Nat Protoc. 2017;12: 255–278. doi: 10.1038/nprot.2016.169 28079879 PMC5540229

[56] Yan Y, Tao H, He J, Huang SY. The HDOCK server for integrated protein–protein docking. Nat Protoc. 2020;15: 1829–1852. doi: 10.1038/s41596-020-0312-x 32269383

[57] Xue LC, Rodrigues JP, Kastritis PL, Bonvin AM, Vangone A. PRODIGY: A web server for predicting the binding affinity of protein-protein complexes. Bioinformatics. 2016;32: 3676–3678. doi: 10.1093/bioinformatics/btw514 27503228

[58] Tina KG, Bhadra R, Srinivasan N. PIC: Protein Interactions Calculator. Nucleic Acids Res. 2007;35. doi: 10.1093/nar/gkm423 17584791 PMC1933215

[59] DeLano WL. The PyMOL Molecular Graphics System, Version 2.3. Schrödinger LLC. 2020. p. http://www.pymol.org. Available: http://www.pymol.org.

[60] Van Der Spoel D, Lindahl E, Hess B, Groenhof G, Mark AE, Berendsen HJC. GROMACS: Fast, flexible, and free. Journal of Computational Chemistry. 2005. pp. 1701–1718. doi: 10.1002/jcc.20291 16211538

[61] Yu Y, Klauda JB. Update of the CHARMM36 United Atom Chain Model for Hydrocarbons and Phospholipids. J Phys Chem B. 2020;124: 6797–6812. doi: 10.1021/acs.jpcb.0c04795 32639155

[62] Martoňák R, Laio A, Parrinello M. Predicting Crystal Structures: The Parrinello-Rahman Method Revisited. Phys Rev Lett. 2003;90: 4. doi: 10.1103/PhysRevLett.90.075503 12633242

[63] Humphrey W, Dalke A, Schulten K. VMD: Visual molecular dynamics. J Mol Graph. 1996;14: 33–38. doi: 10.1016/0263-7855(96)00018-5 8744570

[64] Properties of Antigens in Relation To Responsiveness and Non-Responsiveness. Immunol Toler. 1969; 1–52. doi: 10.1016/b978-1-4832-2727-6.50008–7

[65] Fersht AR, Serrano L. Principles of protein stability derived from protein engineering experiments. Curr Opin Struct Biol. 1993;3: 75–83. doi: 10.1016/0959-440X(93)90205-Y

[66] Matthews BW. Structural and genetic analysis of protein stability. Annual Review of Biochemistry. 1993. pp. 139–160. doi: 10.1146/annurev.bi.62.070193.001035 8352587

[67] Tekewe A, Connors NK, Middelberg APJ, Lua LHL. Design strategies to address the effect of hydrophobic epitope on stability and in vitro assembly of modular virus-like particle. Protein Sci. 2016; 1507–1516. doi: 10.1002/pro.2953 27222486 PMC4972206

[68] Slater K. Structure and stability of the ecosystem. Environ Impact Text. 2003; 1–8. doi: 10.1533/9781855738645.1

[69] McKay MJ, Afrose F, Koeppe RE, Greathouse D V. Helix formation and stability in membranes. Biochimica et Biophysica Acta—Biomembranes. 2018. pp. 2108–2117. doi: 10.1016/j.bbamem.2018.02.010 29447916

[70] Poboinev V V., Khrustalev V V., Khrustaleva TA, Stozharov AN. Stability of alpha-helical and beta-structural blocks in proteins of four structural classes. Proc Natl Acad Sci Belarus, Biol Ser. 2018;63: 391–400. doi: 10.29235/1029-8940-2018-63-4-391-400

[71] Römer RA, Römer NS, Wallis AK. Flexibility and mobility of SARS-CoV-2-related protein structures. Sci Rep. 2021;11. doi: 10.1038/s41598-021-82849-2 33608565 PMC7896093

[72] Tatsi EB, Filippatos F, Michos A. SARS-CoV-2 variants and effectiveness of vaccines: A review of current evidence. Epidemiology and Infection. 2021. pp. 536–544. doi: 10.1017/S0950268821002430 34732275 PMC8632374

[73] Garcia-Beltran WF, Lam EC, St. Denis K, Nitido AD, Garcia ZH, Hauser BM, et al. Multiple SARS-CoV-2 variants escape neutralization by vaccine-induced humoral immunity. Cell. 2021;184: 2372–2383.e9. doi: 10.1016/j.cell.2021.03.013 33743213 PMC7953441

[74] Motozono C, Toyoda M, Zahradnik J, Saito A, Nasser H, Tan TS, et al. SARS-CoV-2 spike L452R variant evades cellular immunity and increases infectivity. Cell Host Microbe. 2021;29: 1124–1136.e11. doi: 10.1016/j.chom.2021.06.006 34171266 PMC8205251

[75] Laurini E, Marson D, Aulic S, Fermeglia A, Pricl S. Molecular rationale for SARS-CoV-2 spike circulating mutations able to escape bamlanivimab and etesevimab monoclonal antibodies. Sci Rep. 2021;11. doi: 10.1038/s41598-021-99827-3 34642465 PMC8511038

[76] McCallum M, Czudnochowski N, Rosen L, Zepeda S, Bowen J, Walls A et al. Structural basis of SARS-CoV-2 Omicron immune evasion and receptor engagement. Science. 2022;375(6583):864–868. doi: 10.1126/science.abn8652 35076256 PMC9427005

[77] Farinholt T, Doddapaneni H, Qin X, Menon V, Meng Q, Metcalf G, et al. Transmission event of SARS-CoV-2 delta variant reveals multiple vaccine breakthrough infections. BMC Med. 2021;19. doi: 10.1186/s12916-021-02103-4 34593004 PMC8483940

[78] Lindstrøm JC, Engebretsen S, Kristoffersen AB, Rø GØI, Palomares ADL, Engø-Monsen K, et al. Increased transmissibility of the alpha SARS-CoV-2 variant: evidence from contact tracing data in Oslo, January to February 2021. Infect Dis (Auckl). 2022;54: 72–77. doi: 10.1080/23744235.2021.1977382 34618665

[79] Kumar V, Singh J, Hasnain SE, Sundar D. Possible Link between Higher Transmissibility of Alpha, Kappa and Delta Variants of SARS-CoV-2 and Increased Structural Stability of Its Spike Protein and hACE2 Affinity. Int J Mol Sci. 2021;22. doi: 10.3390/ijms22179131 34502041 PMC8431609

[80] Zhang L, Jackson CB, Mou H, Ojha A, Peng H, Quinlan BD, et al. SARS-CoV-2 spike-protein D614G mutation increases virion spike density and infectivity. Nat Commun. 2020;11. doi: 10.1038/s41467-020-19808-4 33243994 PMC7693302

[81] Lista MJ, Winstone H, Wilson H, Dyer A, Pickering S, Galao RP, et al. The P681H mutation in the Spike glycoprotein confers Type I interferon resistance in the SARS-CoV-2 alpha (B.1.1.7) variant. bioRxiv. 2021; 2021.11.09.467693. Available: https://www.biorxiv.org/content/10.1101/2021.11.09.467693v1%0Ahttps://www.biorxiv.org/content/10.1101/2021.11.09.467693v1.abstract.

[82] Zhou B, Thao TTN, Hoffmann D, Taddeo A, Ebert N, Labroussaa F, et al. SARS-CoV-2 spike D614G change enhances replication and transmission. Nature. 2021;592: 122–127. doi: 10.1038/s41586-021-03361-1 33636719

